# Nonalcoholic Fatty Liver Disease Is Associated with Aortic Valve Sclerosis in Patients with Type 2 Diabetes Mellitus

**DOI:** 10.1371/journal.pone.0088371

**Published:** 2014-02-05

**Authors:** Stefano Bonapace, Filippo Valbusa, Lorenzo Bertolini, Isabella Pichiri, Alessandro Mantovani, Andrea Rossi, Luciano Zenari, Enrico Barbieri, Giovanni Targher

**Affiliations:** 1 Division of Cardiology, “Sacro Cuore” Hospital, Negrar (VR), Italy; 2 Division of General Medicine “Sacro Cuore” Hospital, Negrar (VR), Italy; 3 Diabetes Unit “Sacro Cuore” Hospital, Negrar (VR), Italy; 4 Section of Endocrinology, Diabetes and Metabolism, Department of Medicine, University and Azienda Ospedaliera Universitaria Integrata of Verona, Verona, Italy; 5 Section of Cardiology, Department of Medicine, University and Azienda Ospedaliera Universitaria Integrata of Verona, Verona, Italy; Institute of Medical Research A Lanari-IDIM, University of Buenos Aires-National Council of Scientific and Technological Research (CONICET), Argentina

## Abstract

**Background:**

Recent epidemiological data suggest that non-alcoholic fatty liver disease (NAFLD) is closely associated with aortic valve sclerosis (AVS), an emerging risk factor for adverse cardiovascular outcomes, in nondiabetic and type 2 diabetic individuals. To date, nobody has investigated the association between NAFLD and AVS in people with type 2 diabetes, a group of individuals in which the prevalence of these two diseases is high.

**Methods and Results:**

We recruited 180 consecutive type 2 diabetic patients without ischemic heart disease, valvular heart disease, hepatic diseases or excessive alcohol consumption. NAFLD was diagnosed by liver ultrasonography whereas AVS was determined by conventional echocardiography in all participants. In the whole sample, 120 (66.7%) patients had NAFLD and 53 (29.4%) had AVS. No patients had aortic stenosis. NAFLD was strongly associated with an increased risk of prevalent AVS (odds ratio [OR] 2.79, 95% CI 1.3–6.1, *p*<0.01). Adjustments for age, sex, duration of diabetes, diabetes treatment, body mass index, smoking, alcohol consumption, hypertension, dyslipidemia, hemoglobin A1c and estimated glomerular filtration rate did not attenuate the strong association between NAFLD and risk of prevalent AVS (adjusted-OR 3.04, 95% CI 1.3–7.3, *p* = 0.01).

**Conclusions:**

Our results provide the first demonstration of a positive and independent association between NAFLD and AVS in patients with type 2 diabetes mellitus.

## Introduction

Non-alcoholic fatty liver disease (NAFLD) has emerged as a public health problem of epidemic proportions worldwide. Up to 30% of adults in the United States and Europe have NAFLD, and the prevalence of this disease is believed to be much higher in people with diabetes [1], [2]. Indeed, the prevalence of NAFLD in patients with type 2 diabetes ranges from approximately 50 to 70% [2]–[4].

Importantly, the global health burden of NAFLD is not only confined to potentially progressive liver disease [2]. Indeed, it has been shown that NAFLD is associated not only with liver-related mortality and morbidity but also with abnormalities of cardiac structure and function [5]–[8] and an increased risk of developing cardiovascular disease (CVD) [9], [10], atrial fibrillation [11] and chronic kidney disease [12].

Recently, a community-based cohort study, involving 2,212 German adult individuals, has shown that NAFLD on ultrasonography is also associated with a greater prevalence of aortic valve sclerosis (AVS), independently of several CVD risk factors [13].

Until recently, AVS was considered an incidental echocardiographic finding of no clinical significance, as it does not significantly obstruct left ventricular outflow. However, AVS shows some epidemiologic and histopathologic similarities to coronary atherosclerosis [14]. In addition, a large number of prospective studies have suggested a strong association between AVS and increased CVD morbidity and mortality in both nondiabetic and type 2 diabetic individuals [15]–[17]. The prevalence of AVS increases progressively with advancing age and is approximately 20% to 30% in subjects aged ≥65 years [15].

To our knowledge, nobody has investigated the association between NAFLD and AVS in people with type 2 diabetes, a group of individuals in which the prevalence of these two diseases is high [2], [3], [17].

Since it is plausible to assume that various biological mechanisms that have been proposed to explain the specific contribution of NAFLD to CVD risk [2], [9], [10], might also be partly implicated in the pathophysiology of AVS, we sought to establish whether NAFLD as diagnosed by ultrasonography (i.e. the most widely used imaging test for diagnosing hepatic steatosis in clinical practice) is associated with AVS in patients with type 2 diabetes.

## Materials and Methods

### Patients

We recruited 180 patients with type 2 diabetes, who were consecutively selected from the whole sample of type 2 diabetic outpatients, who regularly attended the diabetes clinic at the ‘Sacro Cuore’ Hospital of Negrar (Verona) during 2010, after excluding those who did not meet the inclusion criteria for the study.

The exclusion criteria of the study were as follows: (1) patients who had a pre-existing history of myocardial infarction, angina, coronary revascularization procedures, congestive heart failure, moderate-to-severe valvular heart disease, malignancy, cirrhosis and kidney failure, and (2) those with excessive alcohol consumption (i.e., >30 g/day of alcohol for men and >20 g/day for women, respectively), viral hepatitis or other secondary causes of chronic liver disease.

A 12-lead standard resting electrocardiogram, and standard bicycle ergometry were performed in all patients to exclude the presence of silent myocardial ischemia; no patients had any abnormal test results.

The local Ethics Committee of the ‘Sacro Cuore’ Hospital of Negrar approved the study protocol, and all participants gave their written informed consent for participation in this medical research.

### Clinical and Biochemical Measurements

Body mass index (BMI) was calculated by dividing weight in kilograms by the square of height in meters. Waist circumference was measured at the level of the umbilicus. Blood pressure was measured with a mercury sphygmomanometer after patient had been seated quietly for at least 5 minutes. Patients were considered to have hypertension if their blood pressure was ≥140/90 mmHg or if they were taking any anti-hypertensive drugs. Information on daily alcohol consumption, smoking status and current use of medications was obtained from all patients by questionnaire [3].

Venous blood samples were drawn in the morning after an overnight fast. Serum liver enzymes, lipids, complete blood count, creatinine (measured using a Jaffé rate-blanked and compensated assay) and other biochemical blood measurements were determined using standard laboratory procedures (DAX 96; Bayer Diagnostics, Milan, Italy). Normal ranges for serum alanine aminotransferase (ALT) and gamma-glutamyltransferase (GGT) in our laboratory were 10–40 U/l for women and 10–50 U/l for men, respectively. LDL cholesterol was calculated by the Friedewald’s equation, except when plasma triglycerides exceeded 4.55 mmol/l (*n* = 4). Hemoglobin A1c (HbA1c) was measured by an automated high-performance liquid chromatography analyzer (HA-8140; Menarini Diagnostics, Florence, Italy); the upper limit of normal for our laboratory was 5.6%. Glomerular filtration rate (GFR) was estimated by the four-variable Modification of Diet in Renal Disease (MDRD) study equation [18].

### Conventional Echocardiography and Hepatic Ultrasonography

All echocardiographic examinations were performed by a single experienced cardiologist, who was blinded to the subjects’ details. Conventional Doppler echocardiography was used to measure left ventricular (LV) diameters, wall thickness, and mass according to standard criteria [19]. LV end-diastolic and end-systolic volumes and ejection fraction at rest were measured at the apical 2-chamber and 4-chamber views (by modified Simpson’s rule) [19]. Pulsed-wave Doppler was used to measure transmitral peak early diastolic velocity (E), peak late diastolic velocity (A), and E-wave deceleration time (Dte). The presence of AVS was defined as focal or diffuse thickening and calcification of the aortic leaflets without restriction of leaflet motion on echocardiography [17]. A trans-aortic peak instantaneous velocity ≥2.5 m/s was considered as aortic stenosis. Mitral annulus calcification (MAC) was defined by increased echodensity located at the junction of the atrio-ventricular groove and posterior mitral leaflet on the parasternal long-axis, short-axis, or apical 4-chamber view [17].

Hepatic ultrasonography was performed by a single experienced radiologist, who was blinded to the subjects’ details, including echocardiographic data. Hepatic steatosis was diagnosed on the basis of characteristic ultrasonographic features, i.e., evidence of diffuse hyper-echogenicity of the liver relative to the kidneys, ultrasound beam attenuation and poor visualization of intra-hepatic vessel borders and diaphragm. It is known that ultrasonography has good sensitivity and specificity for detecting moderate and severe hepatic steatosis (∼90–95%), but its sensitivity is reduced when the hepatic fat infiltration upon liver biopsy is <30% [20]. A semi-quantitative ultrasonographic scoring for the degree of steatosis (absent, mild, moderate and severe) was assessed by the fall in echo amplitude with depth (rate of posterior beam attenuation), increasing discrepancy of echo amplitude between liver and kidney, and loss of echoes from the walls of the portal veins [7]. The reproducibility of hepatic steatosis scores provided by our single radiologist was very good (intra-observer agreement of 98%) [7].

### Statistical Analysis

Data are expressed as means±SD or proportions. Skewed variables were logarithmically transformed to improve normality prior to analysis. The unpaired Student’s *t*-test and the chi-squared test with Yates’s correction for continuity were used to analyze the differences among the clinical characteristics of patients stratified by the presence of AVS on echocardiography (Table 1). Binary logistic regression analysis was used to study the association between NAFLD and AVS (Table 2). Four forced-entry regression models were performed: an unadjusted model; a model adjusted for age and sex (model 1); a model further adjusted for BMI, smoking history, daily alcohol consumption, hypertension (defined as blood pressure ≥140/90 mmHg and/or anti-hypertensive treatment) and dyslipidemia (defined as LDL-cholesterol >130 mg/dl and/or triglycerides >200 mg/dl and/or HDL-cholesterol <40 mg/dl for women or <35 mg/dl for men and/or lipid-lowering treatment) (model 2); and, finally, a model adjusted for the same variables included in model 2 *plus* duration of diabetes, diabetes treatment (diet *vs*. oral hypoglycemic drugs *vs*. insulin), HbA1c and estimated GFR (model 3). Covariates included in multivariate regression models were chosen as potential confounders based on their biological plausibility or statistical association with AVS in univariate analyses.

**Table 1:** Clinical and biochemical characteristics of participants stratified by aortic valve sclerosis on echocardiography.

	Aortic Valve Sclerosis		
	Absent	Present	*p* value
Sex (male/female, *n*)	98/29	37/16	0.35
Age (years)	68.6±6	69.7±6	0.37
BMI (kg/m^2^)	28.7±4.8	28.5±4	0.94
Waist circumference (cm)	96.8±15	98.6±18	0.57
Current smokers (%)	44.8	39.6	0.70
Diabetes duration (years)	14.1±9	16.9±11	0.09
Systolic blood pressure (mmHg)	143±17	145±15	0.60
Diastolic blood pressure (mmHg)	77±9	78±9	0.82
HbA1c (%)	7.2±1.1	7.6±1.4	<0.05
LDL cholesterol (mmol/l)	2.65±0.8	2.47±0.9	0.19
HDL cholesterol (mmol/l)	1.28±0.3	1.19±0.4	<0.05
Triglycerides (mmol/l)	1.54±0.6	1.76±0.9	<0.05
ALT (U/l)	24±7	28±8	0.21
GGT (U/l)	24±8	29±10	0.18
Estimated GFR (ml/min/1.73 m^2^)	80.8±21	76.8±21	0.24
Dyslipidemia (%)	82.7	86.8	0.36
Hypertension (%)	79.5	92.5	<0.01
Lipid-lowering drugs (%)	79.5	77.3	0.50
Anti-platelet drugs (%)	33.1	37.7	0.45
Oral hypoglycemic drugs (%)	77.2	75.5	0.87
Insulin therapy (%)	40.9	52.8	0.19
ACE-inhibitors (%)	43.3	47.2	0.63
Angiotensin receptor blockers (%)	33.1	45.3	0.12
Calcium-channel blockers (%)	40.2	39.6	0.94
Beta-blockers (%)	23.6	20.7	0.67
Diuretics (%)	42.5	43.4	0.91
LV mass indexed (g/m^2^)	112.9±25	114.7±31	0.68
LV ejection fraction (%)	63.8±8	63.1±8	0.61
E/A ratio	0.72±0.16	0.75±0.14	0.21
MAC (%)	11.8	37.7	<0.005
NAFLD (%)	60.6	81.1	<0.005

Sample size, *n* = 180. Data are means ± SD or proportions. Differences between the two groups were tested by the unpaired Student’s *t*-test (for continuous variables) and the chi-squared test (for categorical variables).

ALT, alanine aminotransferase; BMI, body mass index; GFR, glomerular filtration rate; GGT, gamma-glutamyl-transferase; LV, left ventricular; MAC, mitral annulus calcification.

Hypertension was defined as blood pressure ≥140/90 mmHg and/or anti-hypertensive treatment. Dyslipidemia was defined as LDL-cholesterol >130 mg/dl and/or triglycerides >200 mg/dl and/or HDL-cholesterol <40 mg/dl for women or <35 mg/dl for men and/or lipid-lowering treatment.

**Table 2:** Logistic regression models for NAFLD as a predictor for AVS in patients with type 2 diabetes.

Logistic Regression Models	Odds Ratios (95% CI)	*p* value
NAFLD (yes *vs*. no)		
unadjusted regression model	2.79 (1.3–6.1)	<0.01
adjusted regression model 1	2.67 (1.2–6.0)	0.01
adjusted regression model 2	3.09 (1.3–7.2)	0.01
adjusted regression model 3	3.04 (1.3–7.3)	0.01

Sample size, *n* = 180. Data are expressed as odds ratios ±95% confidence intervals (CI) as assessed by univariable (unadjusted) or multivariable logistic regression analyses.

Other covariates included in multivariable logistic regression models, along with NAFLD, were as follows: **model 1**: age and sex; **model 2:** age, sex, BMI, smoking status, daily alcohol consumption, hypertension (i.e. blood pressure ≥140/90 mmHg and/or anti-hypertensive treatment) and dyslipidemia (i.e. LDL-cholesterol >130 mg/dl and/or triglycerides >200 mg/dl and/or HDL-cholesterol <40 mg/dl for women or <35 mg/dl for men and/or lipid-lowering treatment); **model 3:** adjustment for variables included in model 2 *plus* duration of diabetes, diabetes treatment, HbA1c, and estimated GFR.

All analyses were performed using statistical package IBM-SPSS 19.0 and statistical significance was assessed at the two-tailed 0.05 threshold.

## Results

In the whole sample, NAFLD, AVS and MAC were present in 120 (66.7%), 53 (29.4%) and in 35 (19.4%) patients, respectively. No patients had aortic stenosis (i.e. a trans-aortic peak instantaneous velocity ≥2.5 m/s). No participants had clinical and biochemical characteristics or ultrasonographic findings suggestive of cirrhosis.

Clinical and biochemical characteristics of participants stratified by AVS status on echocardiography are shown in Table 1. Patients with AVS were more likely to have hypertension and MAC, had higher HbA1c, higher triglycerides and lower HDL cholesterol levels than those without AVS. Notably, patients with AVS also had a remarkably higher frequency of NAFLD. Sex, age, BMI, waist circumference, smoking status, diabetes duration, estimated GFR, LDL cholesterol, serum liver enzymes, E/A ratio, LV mass and ejection fraction and use of medications (i.e. hypoglycemic, lipid-lowering and anti-platelet drugs) were not significantly different between the groups. No significant differences were also found in the use of ACE-inhibitors, angiotensin receptor blockers or other classes of anti-hypertensive agents between the two groups of patients.

As expected, when patients were stratified by NAFLD status, those with NAFLD (*n* = 120) were older (69.7±6 *vs*. 67.5±6 years, *p*<0.01), had a longer duration of diabetes (16.5±10 *vs*. 11.9±9 years, *p*<0.01) and were more likely to be centrally obese and hypertensive than those without NAFLD (*n* = 60). NAFLD patients also had significantly (*p*<0.05 for all) lower HDL cholesterol levels, higher serum triglycerides and higher serum liver enzymes (ALT level: 29±9 *vs*. 24±8 U/l; GGT level: 31±9 *vs*. 23±8 U/l), although the majority of our patients had serum liver enzymes within the normal range. In addition, patients with NAFLD had a lower E/A ratio and a remarkably greater frequency of AVS than their counterparts without NAFLD (35.8 *vs*. 16.7%, *p*<0.001). No significant differences were found in sex, smoking, LDL cholesterol, estimated GFR, LV mass, LV ejection fraction, use of medications and proportion of MAC between the two groups of patients.

**Figure 1:**
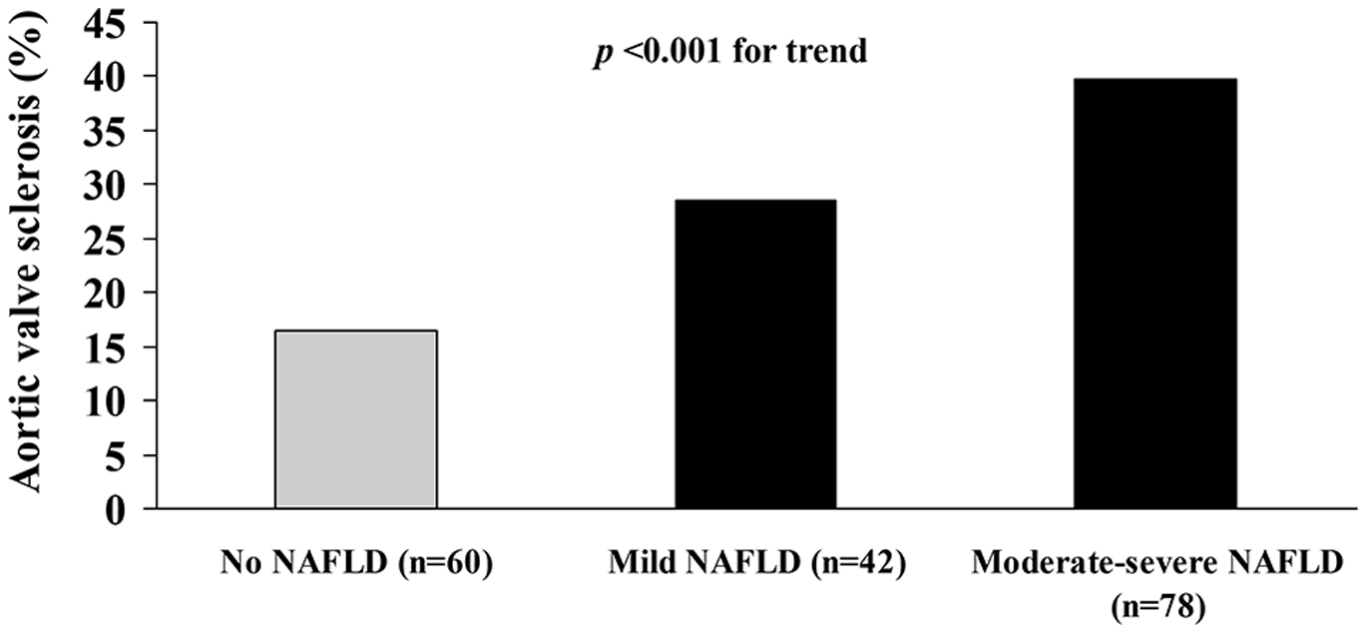
Proportion of type 2 diabetic patients with aortic valve sclerosis (AVS) in relation to the ultrasonographic severity of NAFLD. Data are adjusted for age and sex.

As shown in Figure 1, there was a strong, graded relationship between the presence and severity of NAFLD on ultrasonography (i.e. absent, mild and moderate-to-severe) and the proportion of those with AVS (*p*<0.001 for the trend after adjustment for age and sex).

Table 2 shows the result of the adjustment for potential confounders on the relationship between NAFLD and AVS. In univariate regression analysis (unadjusted model), NAFLD was significantly associated with ∼3-fold increased risk of prevalent AVS (OR 2.79, 95% CI 1.3–6.1, *p*<0.01). After adjustment for age and sex (model 1), NAFLD maintained a significant association with AVS. Further adjustments for BMI, smoking, alcohol consumption, hypertension, dyslipidemia (model 2), and other diabetes-related variables (model 3) did not appreciably weaken the strong association between NAFLD and the risk of AVS. Notably, in this latter regression model, the presence of hypertension (adjusted OR 2.52, 95% CI 1.1–8.2, *p*<0.05) and lower estimated GFR (adjusted OR 1.03, 95% CI 1.01–1.06, *p*<0.05) were also independently associated with AVS.

Almost identical results were found when blood pressure values and plasma lipids (LDL-cholesterol, triglycerides and HDL-cholesterol) were included as continuous measures (instead of categorical variables) in regression models 2 and 3 or when the results were additionally adjusted for serum aminotransferases (data not shown).

Interestingly, results remained unchanged when we excluded from analysis those with MAC on echocardiography (*n* = 35). Again, NAFLD was closely associated with AVS (OR 7.21, 95% CI 2.1–25.1, *p*<0.005).

Conversely, when we repeated the above logistic regression analyses using the presence of MAC as dependent variable (instead of AVS), NAFLD was not significantly associated with MAC in univariate analysis (OR 1.32, 95% CI 0.6–2.9, *p* = 0.52).

## Discussion

Our novel finding was that NAFLD as detected by ultrasonography was strongly associated with AVS, i.e. an emerging risk factor for adverse CVD outcomes, in patients with type 2 diabetes without pre-existing history of ischemic heart disease, valvular heart disease, chronic liver diseases or excessive alcohol consumption. Notably, and most importantly, this association remained statistically significant even after adjusting for several established CVD risk factors and diabetes-related variables. In addition, there was a strong, graded relationship between the ultrasonographic severity of NAFLD and the proportion of those with AVS. Conversely, no association was found between NAFLD and the presence of MAC on echocardiography.

Our findings expand to patients with type 2 diabetes the recent observations reported by the investigators of the Study of Health in Pomerania [13]. Interestingly, in such population-based cohort study of 2,212 German men and women aged 45 to 81 years, the investigators have shown that NAFLD on ultrasonography was significantly associated with an increased risk of prevalent AVS (adjusted OR 1.32, 95% CI 4–66; *p* = 0.02) even after controlling for several established CVD risk factors and potential confounders, including estimated GFR, C-reactive protein, serum ferritin, and white blood cells [13].

Another interesting finding of our study that corroborates previously published observations [2]–[4], is that the frequency of NAFLD on ultrasonography was high among patients with type 2 diabetes (i.e. approximately two-thirds of our patients had NAFLD), and that the majority of those with NAFLD had serum liver enzyme levels within the “normal” reference range. This further suggests that serum liver enzyme levels are insensitive markers for the detection of NAFLD, and that the “normal” reference values for serum liver enzymes currently used to exclude NAFLD need to be revised [1], [2].

Collectively, therefore, the present results provide further strong evidence that NAFLD and AVS are two inter-related pathologic conditions, in part independent from traditional CVD risk factors and diabetes-related variables. Although further studies are certainly needed to corroborate our findings, these results support the possibility that the presence of AVS might represent an additional link to increased CVD risk observed among patients with NAFLD, and further emphasize the clinical importance of evaluating the global CVD risk in this group of patients [1], [2], [9], [10]. It is known that AVS is a powerful predictor of adverse CVD outcomes, independently of traditional risk factors, both in patients without diabetes and in those with type 2 diabetes [15]–[17].

The underlying mechanisms responsible for the observed association between NAFLD and AVS remain speculative and require further study. Speculatively, the most plausible explanation for our findings is that the association between NAFLD and AVS is a simple epiphenomenon of shared CVD risk factors and co-morbidities or, alternatively, a marker of ectopic fat deposition in other organs such as the myocardium or pericardium. For instance, Mahmod *et al*. have recently reported that myocardial steatosis as measured by proton magnetic resonance spectroscopy (^1^H-MRS) was common in nondiabetic and diabetic patients with severe aortic stenosis, and was significantly associated with impaired LV systolic function [21]. Rijzewijk *et al*. also found that myocardial steatosis was a strong predictor of LV dysfunction in people with type 2 diabetes [22]. Interestingly, the same investigators also showed that higher myocardial fat content was significantly associated with higher intra-hepatic fat content as detected by ^1^H-MRS [22]. Again, in a small study of uncomplicated type 2 diabetic men without ischemic heart disease, Rijzewijk *et al*. found that compared with those with lower intra-hepatic fat content, patients with higher intra-hepatic fat content on ^1^H-MRS had significantly decreased myocardial perfusion, glucose uptake and high-energy phosphate metabolism [23]. Although preliminary evidence also suggests that increased pericardial fat volume was significantly associated with the presence of coronary heart disease, and independently predicted subsequent development of CVD events in the community [24], [25], no published data are currently available about the relationship between increased pericardial fat, NAFLD and AVS. Finally, since in our study there was a strong, graded relationship between the presence and severity of NAFLD and AVS that was independent of multiple CVD risk factors, it is also plausible to assume that NAFLD, especially its necro-inflammatory form (NASH), is not only a simple marker of AVS in type 2 diabetes but also may be, at least in part, involved in its pathogenesis. This process may occur through the contribution of NAFLD *per se* to systemic and hepatic insulin resistance and/or through the systemic release of several pathogenic mediators from the steatotic and inflamed liver, such as increased reactive oxygen species, advanced glycation end products, C-reactive protein, plasminogen activator inhibitor-1, transforming growth factor-beta and other pro-inflammatory, pro-coagulant and pro-fibrogenic factors. Notably, several case-control studies have shown that these potential mediators of vascular injury are remarkably higher in patients with NAFLD than in those without the disease [2], [9], [26], [27].

Thus, although the potential role of non-cirrhotic NAFLD in the pathophysiology of AVS requires further testing and confirmation in larger studies, we believe that this is a promising field of research to explore, and that the pathways that involve the specific contribution of NAFLD to systemic insulin resistance, chronic inflammation and hypercoagulation might provide a potential therapeutic target for the treatment and prevention of AVS in people with NAFLD.

Our study has some important limitations. First, the design of our study is cross-sectional and cannot determine any causal or temporal relationship between NAFLD and AVS. Second, the diagnosis of NAFLD was based on ultrasonography and the exclusion of other causes of chronic liver diseases but was not confirmed by liver biopsy (i.e. the only proven method to distinguish simple steatosis from NASH), which would be unethical to perform in our patients who had normal or only mildly elevated serum liver enzymes. Conversely, ultrasonography is by far the most common way of diagnosing NAFLD in clinical practice, and has a good sensitivity and specificity in detecting moderate and severe hepatic steatosis. Indeed, it has been reported that the presence of >30% fat on liver biopsy is optimal for ultrasound detection of steatosis, whereas ultrasonography is not totally sensitive, particularly when hepatic fat infiltration is less than 30% [20]. Thus, although some non-differential misclassification of NAFLD on the basis of ultrasonography is likely (i.e., some of the diabetic control patients could have underlying NAFLD despite normal serum liver enzymes and negative ultrasonography examination), this limitation would serve to attenuate the magnitude of our effect measures toward null; thus, our results can probably be considered as conservative estimates of the association between NAFLD and AVS. However, given the cross-sectional nature of our study, it is also important to note that the temporal profiles of the AVS severity (that usually progresses in many patients over time) and the amount of hepatic fat infiltration in NAFLD (that usually improves or resolves in many patients who progress to cirrhosis) may substantially differ over time. Finally, we cannot exclude residual confounding as a result of unmeasured (e.g. plasma inflammatory biomarkers) or unknown or unmeasured risk factors (e.g. factors that may promote aortic valve calcification).

Notwithstanding these limitations, our study has several important strengths, including the relatively large number of participants who were free of diagnosed ischemic heart disease, the diagnosis and severity of hepatic steatosis by ultrasonography (which was performed by a single experienced radiologist), the exclusion of patients with cirrhosis (we believe that the inclusion of patients with such complication would have confounded the interpretation of the data), the completeness of the dataset, and the ability to adjust for multiple established CVD risk factors and potential confounders.

In conclusion, this is the first study to demonstrate that NAFLD is closely associated with an increased prevalence of AVS in patients with type 2 diabetes, and that this association is independent of several established CVD risk factors and diabetes-related variables. Further studies are needed to corroborate these findings in independent samples, and to better elucidate the responsible mechanisms for this association. It is also important to determine whether improvement in NAFLD (or future treatments for NAFLD) will ultimately delay or prevent the development and progression of AVS.
